# A Three-Dimensional Porous Ag/C/Sodium Alginate@Polyurethane Sponge for Efficient Solar-Driven Seawater Desalination

**DOI:** 10.3390/nano16140864

**Published:** 2026-07-14

**Authors:** Yingying Yue, Rou Zeng, Yingfei Wang, Jiaqi Hu, Chengxin Zhang, Yu Li, Weijie Wei, Wubo Wan, Zaoxi Li, Yaqin Shi

**Affiliations:** 1 Yazhou Bay Innovation Institute, College of Food Science and Engineering, Hainan Tropical Ocean University, Sanya 572022, China; zengrou2025@163.com (R.Z.); yfwang@hntou.edu.cn (Y.W.); 18776555069@163.com (J.H.); jh664344138@163.com (C.Z.); m19814788056@163.com (Y.L.); wanwubo@163.com (W.W.); m13086077857@163.com (Z.L.); 2 School of Chemistry and Chemical Engineering, Hainan University, Haikou 570228, China; m15759114098@163.com

**Keywords:** used coffee powder, seawater desalination, composite hydrogel, photothermal material

## Abstract

Carbonized coffee grounds exhibit excellent light absorption properties, while sodium alginate hydrogel is characterized by good biocompatibility, low toxicity, and low cost, rendering it suitable for the fabrication of multi-functional photothermal materials. In this study, polyurethane (PU) sponge and sodium alginate were used as the matrix, onto which coffee ground-derived carbon and silver nanoparticles (AgNPs) were loaded to construct a three-dimensional (3D) porous network. A high-performance, recyclable photothermal-steam conversion composite hydrogel sponge was successfully prepared via a simple drying method. The microstructure, physicochemical stability, mechanical properties, and photothermal performance of the composite sponge were systematically characterized, and the interfacial interaction mechanism between the components was clarified. The results showed that stable interactions were formed between coffee ground-derived carbon and sodium alginate, which regulated the water evaporation rate during the photothermal process. The introduction of AgNPs not only enhanced the mechanical strength of the composite material but also achieved a high photothermal conversion efficiency of up to 92.32%. This study broadens the application prospects of waste coffee ground-derived carbon in the field of photothermal conversion.

## 1. Introduction

Globally, freshwater resources are becoming increasingly scarce, and the extraction of salt ions from seawater to produce freshwater has emerged as one of the crucial approaches to ensuring water resource supply [1]. Solar-driven interfacial evaporation (SDIE) is recognized as a sustainable seawater desalination technology that can effectively alleviate freshwater shortages in several countries [2]. In SDIE technology, solar energy serves as the primary energy source; seawater reaches the light-absorbing layer through specific water transport channels and undergoes phase transition to produce usable freshwater upon solar irradiation [3]. The performance of SDIE systems is mainly determined by the design and integration of photothermal materials, which involves four key stages: light absorption [4], thermal conversion [5], water transport [6], and heat conduction [7].

Hydrogels are materials composed of hydrophilic polymer networks [8]. They exhibit responsiveness to environmental changes, such as variations in pH [9], salt concentration [10], and temperature [11], and possess high biocompatibility [12]. The network structure of hydrogels can be tailored by adjusting experimental conditions and cross-linking with other materials, thus realizing the optimization of material properties while preserving their intrinsic characteristics. To endow single-component hydrogels with more excellent and versatile properties, numerous studies have prepared multi-functional composite hydrogels by combining hydrogels with other substances. These composite hydrogels have been widely applied in biomaterials [13], medicine [14], the food industry [15], personal care products [16], agriculture [17], and environmental remediation [18]. Sodium alginate, a natural polymer isolated from brown algae, is one of the common raw materials for hydrogel preparation [19]; it can form three-dimensional network-structured hydrogels via thermogelation, ionic cross-linking, covalent cross-linking, cell cross-linking, and free radical polymerization [20].

SDIE technology can be integrated with biological waste, which not only mitigates the environmental impact of solid waste but also enables freshwater production in a more cost-effective manner. With the growing popularity of coffee, the yield of coffee beans has increased steadily. A large quantity of coffee by-product, namely coffee grounds, is generated during the preparation of a single cup of coffee. Coffee grounds are typically discarded in powder form, with lignin as their main structural component [21]. Lignin is a complex three-dimensional network natural polymer [22] that contains abundant functional groups such as phenolic hydroxyl, alcoholic hydroxyl, and carbonyl groups in its structure [23]. Compared with macro-scale silver materials, silver nanoparticles (AgNPs) endow materials with a higher specific surface area and an increased density of surface active sites, and they possess unique optical properties [24] and can synergistically exert antibacterial activity upon light irradiation [25]. For these reasons, AgNPs have been extensively used in the fabrication of composite materials.

This study aims to realize the high-value utilization of waste coffee grounds and achieve efficient and economical seawater desalination. Composite hydrogels were prepared using coffee powder carbon material (CPCM), with polyurethane (PU) sponge and sodium alginate (SA) as the matrix materials and CPCM loaded as the desalination agent. Silver nanoparticles were introduced as comonomers to fabricate a photothermal-steam conversion material. A solar-driven interfacial evaporator was constructed by using cotton swabs as water transport channels, non-woven fabric as the medium, and Ethylene vinyl acetate (EVA) foam as the support for the prepared photothermal-steam conversion material. The photothermal performance, water evaporation performance, recyclability, and antifouling property of the evaporator were tested under one sun illumination. The primary objective of this work is to fabricate a composite hydrogel sponge-based solar-driven interfacial evaporator with low water evaporation enthalpy, high thermal efficiency, stable water supply, and excellent structural and performance stability.

## 2. Materials and Methods

### 2.1. Materials and Chemicals

The materials used were: waste coffee grounds (obtained by air-drying the residue after coffee extraction from a local coffee shop, Sanya, China), alkaline lignin (purchased from Xilong Scientific Co., Ltd., Shantou, China), non-woven fabric (Qingdao Qingfeng Nonwoven Technology Co., Ltd., Qingdao, China), water-washed cotton swabs, experimental water (deionized water), PU sponge, evaporation solution (NaCl solution, 35 g/L), circular cutter (purchased from local online hardware supplier, Sanya, China), silver nitrate, ammonia water, calcium chloride, sodium chloride, dilute hydrochloric acid, and sodium hydroxide (all purchased from Xilong Scientific Co., Ltd., Shantou, China). All figures in this manuscript were plotted using Origin 2021 (OriginLab Corporation, Northampton, MA, USA). XPS spectral data were fitted and analyzed via XPSPeak 4.1 software(https://xpspeak.software.informer.com/, accessed on 5 July 2026).

### 2.2. Synthesis of CPCM/SA

**Figure 1:**
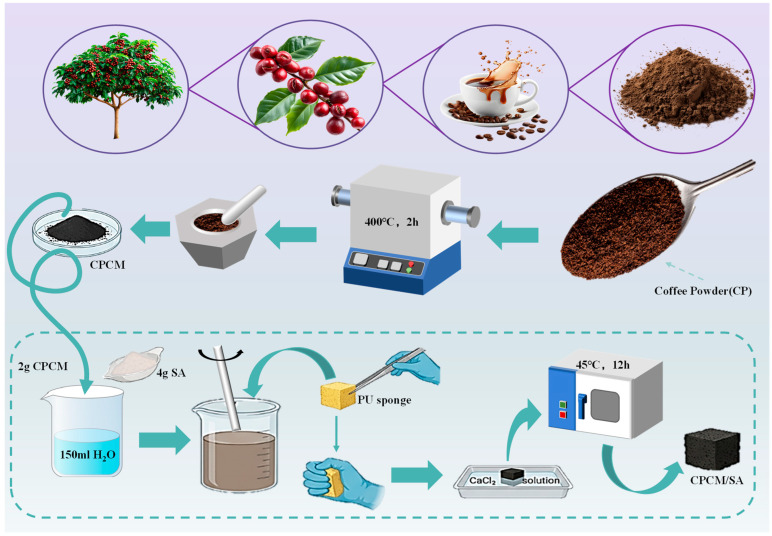
Schematic illustration of the preparation process of CPCM/SA.

An appropriate amount of coffee powder (CP) was placed in a porcelain boat and carbonized in a tube furnace at 400 °C for 2 h. After carbonization, the product was taken out, ground and sieved through a 0.0075 mm mesh sieve to obtain coffee powder carbon material (CPCM). Two portions of sodium alginate (SA, 4 g each) were separately added to 150 mL of deionized water, and 2 g of CPCM was added to one of the two SA aqueous solutions. The two mixtures were respectively stirred on a constant temperature magnetic stirrer(IKA Works, Guangzhou, China) at a rotation speed of 15 r/min and a temperature of 65 °C for 4 h to prepare the pristine SA solution and CPCM/SA modified solution. The raw polyurethane (PU) sponges were fully immersed in the pristine SA solution and CPCM/SA modified solution, respectively. After repeated extrusion to ensure the complete infiltration of the sponges, the samples were taken out and left to stand. Calcium chloride (CaCl_2_) was then added to fabricate the composite hydrogels via an ionic cross-linking method, during which the modified solutions were immobilized in the PU sponges. Finally, the samples were dried for 12 h until no moist feeling was observed on the sponge surface, yielding the SA three-dimensional (3D) sponge skeleton and CPCM/SA composite hydrogel sponge (Figure 1).

### 2.3. Synthesis of AgNPs-CPCM/SA

First, 0.5 g of alkaline lignin was added to 25 mL of deionized water and stirred at room temperature until the lignin was fully mixed with the aqueous solution. Separately, 200 mg of silver nitrate (AgNO_3_) and 5 mL of ammonia solution were added to 20 mL of deionized water successively to prepare an ammoniated AgNO_3_ solution (silver-ammonia complex solution). At this stage, silver ions (Ag^+^) formed stable and soluble complex cations [Ag(NH_3_)_2_]^+^ with ammonia molecules (NH_3_). Compared with free Ag^+^, [Ag(NH_3_)_2_]^+^ is milder and has a lower reduction potential, which makes the reduction reaction more controllable and avoids the rapid reduction of Ag^+^ into large silver particles or silver mirrors.

The previously prepared lignin aqueous solution was added dropwise to the ammoniated AgNO_3_ solution and stirred uniformly. Lignin acted as a reducing agent, and Ag^+^ gained one electron from the phenolic hydroxyl groups in lignin to be reduced, thereby generating silver nanoparticles (AgNPs). An excess amount of sodium chloride (NaCl) solution was then added to remove oxidized silver ions, resulting in lignin-encapsulated AgNPs. Groups such as carbonyl and ether bonds in lignin molecules were adsorbed on the surface of AgNPs through physical or chemical interactions to form an encapsulated structure. This lignin protective layer exerted a strong steric hindrance effect: when two lignin-encapsulated AgNPs particles approached each other, the mutual repulsion between the lignin layers prevented direct contact and agglomeration of the particles, thus ensuring the long-term stability of AgNPs.

Subsequently, 4 g of sodium alginate (SA), 2 g of CPCM, and 50 mL of lignin-AgNP composite aqueous solution were placed in a beaker, followed by the addition of an appropriate amount of deionized water. The mixture was fully stirred on a constant temperature magnetic stirrer at 65 °C for 4 h to obtain the modified AgNPs-CPCM/SA solution. A polyurethane (PU) sponge was immersed in the modified solution; after complete infiltration through repeated extrusion, the sponge was taken out and left to stand. Calcium chloride (CaCl_2_) cross-linking agent was added, and the sample was dried for 8 h to finally obtain the AgNPs-CPCM/SA composite hydrogel sponge.

### 2.4. Characterizations

The morphologies of SA, CPCM/SA, and AgNPs-CPCM/SA were observed using a field emission scanning electron microscope (SEM, Thermoscientific Verios G4 UC, Hillsboro, OR, USA), respectively. The surface elemental compositions of SA, CPCM/SA, and AgNPs-CPCM/SA were analyzed by an energy-dispersive X-ray spectrometer (EDS, Oxford INCA Energy 200, Abingdon, UK). The chemical bonds and functional groups of SA, CP, CPCM/SA, and AgNPs-CPCM/SA were characterized using a Fourier transform infrared spectrometer (FTIR, Nicolet iS50, Madison, WI, USA) with a scanning range of 500–4000 cm^−1^. The light absorption capacities of SA, CPCM/SA, and AgNPs-CPCM/SA were measured via an ultraviolet/visible/near-infrared (UV/Vis/NIR) spectrophotometer (PerkinElmer Lambda 950, Waltham, MA, USA). A handheld thermal imaging camera was used to record the temperature changes in SA, CPCM/SA, and AgNPs-CPCM/SA within 6 min, with temperature measurements taken and recorded every minute. The water contact angles (WCA) of SA, CPCM/SA, and AgNPs-CPCM/SA were tested using an optical contact angle goniometer (DataPhysics Oca25, Filderstadt, Germany), respectively.

### 2.5. Calculation of Water Evaporation Rate

We estimated the capillary action intensity of the evaporator through the water evaporation rate. A xenon lamp light source was used to simulate sunlight (light intensity adjusted to 1 kW/m^2^) in the seawater evaporation experiment, and a 35 g/L NaCl solution was prepared to simulate seawater. The self-constructed simple seawater desalination device was placed under the xenon lamp for irradiation, and the light intensity was measured by an optical power meter. During the experiment, the ambient temperature was 24 °C and the humidity was 77%. The device was irradiated continuously for 4 h, and its weight was measured with an electronic analytical balance every hour. The evaporation rate was calculated by recording the changes in mass loss and irradiation time. In the process of calculating the evaporation efficiency of the evaporator, it is necessary to consider the natural evaporation of water, so the water evaporation rate without sunlight irradiation should be measured and deducted when calculating the total water evaporation rate. The evaporator was irradiated under a xenon lamp, the light intensity was determined by an optical power meter, and the changes in mass loss and irradiation time were recorded to calculate the evaporation rate. The evaporation rate m (kg·m^−2^·h^−1^) can be calculated by Formula (1):

(1) $$\overset{\cdot }{m}=\frac{∆m}{S\times ∆t}$$

where *ṁ* is the water mass loss of the evaporator (kg·m^−2^·h^−1^);

*S* is the evaporation area of the evaporator (m^2^); and Δ*t* is the evaporation time (h).

All experiments were repeated in at least three parallel tests under the same environmental conditions.

### 2.6. Calculation of Water Evaporation Efficiency

To evaluate the water evaporation efficiency of photothermal conversion materials, the solar-to-steam conversion efficiency (*η*) was calculated using the following Formula (2) [26]:

(2) $$\eta =\frac{\overset{\cdot }{m}\times (h_{lv}+W_{\mathrm{least}})}{Q_{solar}}$$

where *ṁ* is the evaporation rate (kg·m^−2^·h^−1^);

*Q_solar_* is the light intensity of the xenon lamp per unit time (1 kW·m^−2^·h^−1^ = 3600 kJ·m^−2^·h^−1^);

$h_{lv}$ is the total enthalpy change in the liquid phase transforming into the gas phase (kJ·kg^−1^);

*W_least_* refers to the theoretical minimum energy required to remove salts from the salt solution and produce pure water. For a 35 g/L NaCl solution, the energy required to separate it into pure water and solid salt is 10.75 kJ, which is equivalent to 10.39 kJ per kilogram of pure water. 10.39 kJ/kg is regarded as the approximate energy consumption value for the 35 g/L NaCl solution [27].

The natural dark evaporation of water was measured and subtracted during the calculation to eliminate environmental interference.

### 2.7. Calculation of Water Evaporation Enthalpy

The equivalent evaporation enthalpy was measured in a sealed, dark and thermally insulated environment to avoid extra heat gain from ambient light and surroundings. To determine the equivalent evaporation enthalpy of the photothermal conversion material, an evaporator with a pure water sample and an evaporator with 35 g/L NaCl solution were constructed and placed together in a dark and sealed environment. By monitoring the mass loss of both within 12 h and combining the principle of energy conservation, the equivalent evaporation enthalpy of the hydrogel was calculated using the Formulas (3) and (4):

(3) $$h_{lv}=h_{ee,eva,T1}+h_{sh,eva,T1}$$

(4) $$h_{ee,eva,T1}=\frac{{\overset{\cdot }{m}}_{water}}{{\overset{\cdot }{m}}_{eva}}\times h_{ee,water,T1}$$

Among them, $h_{lv}$ is the total enthalpy change from water to steam (kJ·kg^−1^);

${\overset{\cdot }{m}}_{water}$ and ${\overset{\cdot }{m}}_{eva}$ are the dark-field evaporation rates of the pure water evaporator and the 35 g/L NaCl solution evaporator, respectively (kg·m^−2^·h^−1^);

$h_{ee,water,T1}$ and $h_{ee,eva,T1}$ are the evaporation enthalpies of pure water and the evaporator, respectively (kg·m^−2^·h^−1^).

$h_{sh,eva,T1}$ is the sensible heat of evaporation, which refers to the heat absorbed by the liquid when it is heated from the initial temperature to the boiling point, the Formula (5) is:

(5) $$h_{sh,eva,T1}=c_{p}\times ∆t$$

Among them, $c_{p}$ is the specific heat capacity of water (4.186 kJ·kg^−1^), the boiling point of water is 100 °C, and the initial temperature of water is taken as 25 °C; thus, $h_{sh,eva,T1}$ is 313.95 kJ/(kg·°C).

### 2.8. Photocatalytic Degradation Performance Test

Typical organic dyes methylene blue (MB) and rhodamine B (RhB) were selected as simulated pollutants to evaluate the visible-light photocatalytic performance of AgNPs-CPCM/SA composite sponges. MB and RhB simulated wastewater solutions with an identical concentration of 20 mg/L were prepared. 40 mL of each dye solution was transferred into quartz reaction vessels, followed by the addition of 10 mg of AgNPs-CPCM/SA powder. The mixture was first stirred in the dark for 30 min to achieve adsorption–desorption equilibrium between the composite material and dye molecules. A 300 W xenon lamp was then turned on to provide visible-light irradiation. At predetermined time intervals, 3 mL of the reaction solution was collected for subsequent testing. The absorbance variation in the filtrate was measured by ultraviolet-visible spectrophotometer. The characteristic absorption peak intensities of MB at 664 nm and RhB at 554 nm were recorded to assess the photocatalytic removal capacity of the prepared material.

The pseudo-first-order kinetic equation was adopted to quantitatively evaluate the photocatalytic degradation rate of MB and RhB:

(6) $$-ln\frac{A_{t}}{A_{0}}=kt$$

where $A_{0}$ and $A_{t}$ represent the absorbance of pollutant solution at initial time and reaction time t, respectively; k is the apparent pseudo-first-order rate constant.

## 3. Results and Discussion

### 3.1. Microscopic Morph

**Figure 2:**
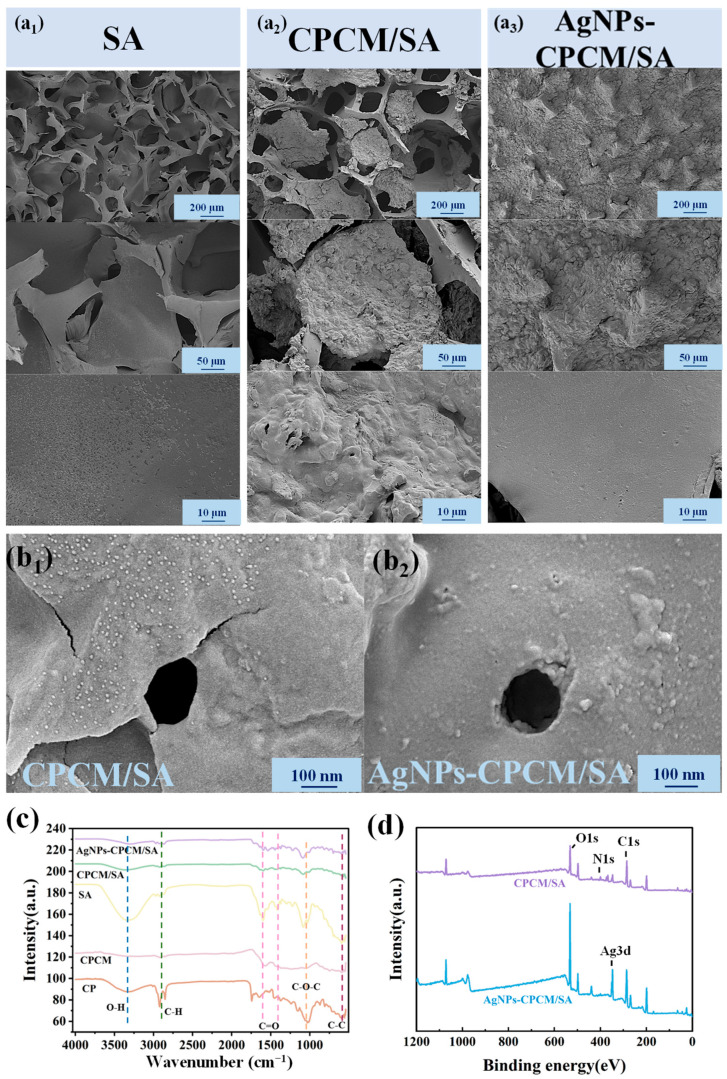
(**a_1_**–**a_3_**) Micron-scale SEM images of SA, CPCM/SA and AgNPs-CPCM/SA; (**b_1_**,**b_2_**) nanoscale SEM images of CPCM/SA and AgNPs-CPCM/SA; (**c**) the FTIR images of CP, CPCM, SA, CPCM/SA, AgNPs-CPCM/SA; (**d**) the XPS images of CPCM/SA and AgNPs-CPCM/SA.

Observe and analyze the microscopic morphologies of SA, CPCM/SA, and AgNPs-CPCM/SA using SEM. As can be seen from Figure 2(a_1_), there are particles on the three-dimensional network skeleton, which retains the pore characteristics of the base material and increases the surface roughness. It is speculated that the sodium ions in the SA structure successfully undergo ionization displacement reaction with calcium in the aqueous solution to generate cross-linking sites, forming a stable gel loaded on the PU sponge skeleton. From Figure 2(a_2_), it can be observed that the composite hydrogel formed by the physical blending of the hydrogel matrix SA and the biomass-based compound CPCM enters the pores of the three-dimensional network matrix. After the loading of CPCM, the macroscopic morphology of the sponge matrix changes. The CPCM powder uniformly covers the sponge channels to form a modified layer, which provides more binding sites for the subsequent loading of AgNPs. At the same time, local gelation occurs, the rheological properties decrease, and the loaded material is not easy to lose. Ca^2+^ plays a crucial role in improving the mechanical properties of alginate hydrogels. CaCl_2_ undergoes ionic cross-linking with Na^+^ in sodium alginate. Ca^2+^ is adsorbed due to the electrostatic attraction between it and the carboxyl groups on the SA skeleton, forming an insoluble gel structure [28]. No covalent bonds are involved in this gelation process, and the interior of the hydrogel is mainly cross-linked through relatively weak interactions (electrostatic interactions, hydrogen bonding, hydrophobic interactions, etc.), so it may have the disadvantages of low mechanical strength and easy depolymerization [29]. CPCM improves these problems of Ca^2+^ alginate hydrogels, and no chemical cross-linking agents are introduced during the preparation process, so it is non-toxic. After loading AgNPs, the surface of the 3D skeleton becomes rougher with accumulated particles. The change in surface morphology may affect the optical properties of the composite material. There are a large number of hydroxyl and carboxyl groups on the SA three-dimensional network skeleton, which is conducive to the mild embedding and immobilization of silver nanoparticles. AgNPs are uniformly dispersed in the three-dimensional network skeleton, which may be due to the repulsive force between two adjacent lignin-encapsulated AgNP particles, making them not easy to bond. Figure 2b shows that CPCM/SA and AgNPs-CPCM/SA contain nanoscale pores, which are conducive to the rapid transport of water molecules during the photothermal evaporation process, and water molecules are not easy to form agglomerates, reducing the time of the water-steam phase transition process.

Fourier transform infrared spectroscopy (FTIR) was adopted to characterize the molecular structures of raw monomer, substrate sponge and composite hydrogel sponge. Measurements were performed within the mid-infrared wavenumber range of 5004000 cm^−1^, where fundamental vibrational signals of most organic and inorganic species can be detected. As displayed in Figure 2c, distinguishable differences in functional group signals are observed between SA and CP. SA presents typical FTIR fingerprints characteristic of organics bearing abundant carboxyl and hydroxyl moieties. For the CP sample, prominent C–H stretching vibrations are detected at 2843–3000 cm^−1^; the intensity of these C–H absorption bands declines after carbonization and further composite fabrication. The attenuation of CH peaks can be partially ascribed to the cleavage of low-energy C–H bonds during thermal carbonization, accompanied by the decomposition of aliphatic and unsaturated hydrocarbon fragments derived from coffee grounds, which simultaneously eliminates H– and O–containing heteroatomic groups. After carbonization, the CPCM framework is dominated by C–C and C=C carbon skeletons alongside reduced C–H species. Combined with the rough surface morphology confirmed by SEM (Figure 2(a_2_)), such a carbon-rich surface structure may potentially suppress energy dissipation and optimize light harvesting capacity. Moreover, the elevated surface carbon proportion is beneficial to the adsorption capacity of the sponge, and reduces the risk of secondary water pollution induced by leachable organic fragments.

The broad O–H stretching absorption band located at 3200–3400 cm^−1^ of pristine SA and CP exhibits decreased absorbance intensity after carbonization, which can be attributed to the dehydration reaction that consumes partial hydroxyl and carboxyl groups. The overall spectral profiles of CP and CPCM resemble that of raw SA. A distinct absorption signal at 2190 cm^−1^ is observed for CP, whereas the absorbance intensities of other characteristic peaks decline markedly. The O–H and C=O peak intensities of SA and CPCM/SA are suppressed or nearly vanish upon composite fabrication, implying the consumption or coordination interaction of hydroxyl and carboxyl groups during cross-linking. Detectable C–H vibrational peaks remain in all samples, revealing that the organic backbone is not fully decomposed after compositing and carbonization. A slight shift in the carboxyl absorption band is recorded after AgNPs immobilization, confirming intermolecular interaction between AgNPs and the sodium alginate matrix; such interfacial interaction may alter the cross-linking state of the composite network.

The mid-infrared region can be classified into the characteristic frequency region (1330–4000 cm^−1^) and fingerprint region (500–1330 cm^−1^). The fingerprint region is sensitive to minor variations in molecular structures despite its weak characteristic absorption signals. Slight discrepancies of fingerprint-region absorption peaks are identified between CPCM/SA and AgNPs-CPCM/SA, verifying that AgNP loading induces minor adjustments to the molecular structure of the substrate composite.

As displayed in Figure 2d, XPS was utilized to analyze the elemental valence states of CPCM/SA and AgNPs-CPCM/SA composites. Distinct characteristic Ag signals were observed in the XPS survey spectrum of AgNPs-CPCM/SA, which verifies the successful incorporation of silver species into the CPCM/SA matrix.

**Figure 3:**
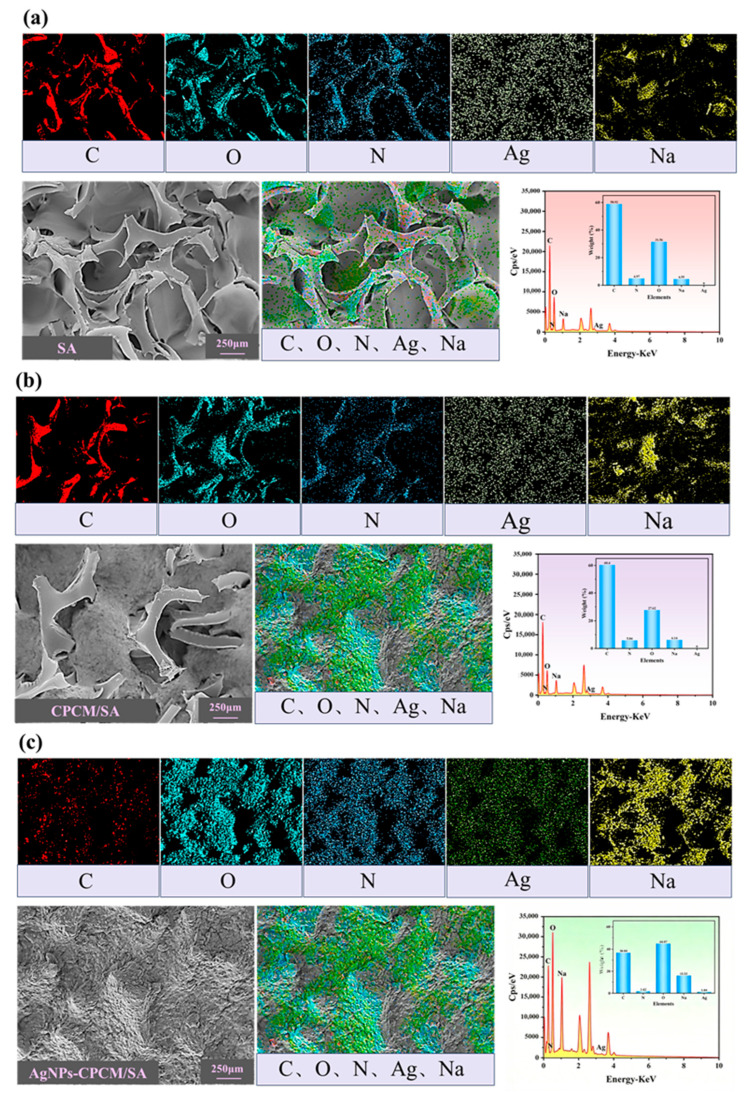
SEM-EDS images and elemental distribution for SA (**a**), CPCM/SA (**b**) and AgNPs-CPCM/SA (**c**).

Further compositional characterization of the composite hydrogel sponge was performed, and the surface element distribution and content of SA, CPCM/SA, and AgNPs-CPCM/SA were analyzed by EDS spectra. Figure 3a shows that the base material mainly contains three elements: C, H, and O, and Na element is introduced by SA. CPCM/SA and AgNPs-CPCM/SA contain five characteristic elements: C, H, O, Na, and Ag. In Figure 3b,c, the Na and Ag elements change from slight dispersion to uniform dispersion. The uniform dispersion and significant increase in the content of Ag element indicate that AgNPs have been successfully decorated on the CPCM particles and present a uniformly distributed state in the sponge matrix. It can be seen that the enrichment of the carbon skeleton enhances light absorption, the densification of the structure reduces heat energy loss, and the reduction in C-H bonds strengthens stability. These three factors jointly promote the performance optimization of coffee grounds-based photothermal materials.

### 3.2. Wettability and Chemical Stability

**Figure 4:**
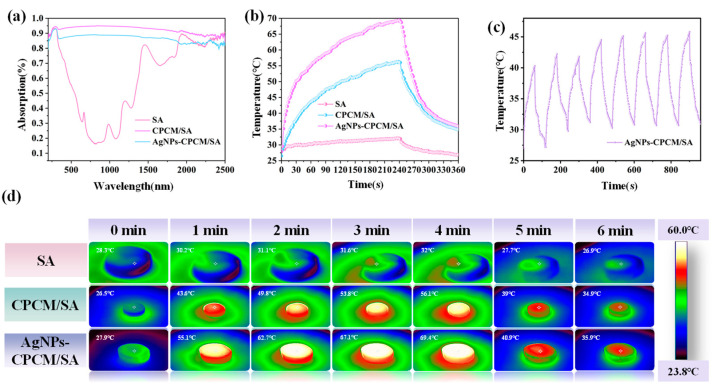
(**a**) Absorption of SA, CPCM/SA and AgNPs-CPCM/SA; (**b**) temperature change during 6 min of heating and cooling under sunlight for SA, CPCM/SA and AgNPs-CPCM/SA; (**c**) temperature change during 8 heating–cooling cycles of AgNPs-CPCM/SA; (**d**) thermal images of 6 min heating and cooling for SA, CPCM/SA and AgNPs-CPCM/SA (The diamond symbol in the contour image represents the central detection point of the sample).

The photothermal conversion process of photothermal conversion materials mainly includes light absorption and light conversion. More heat can be obtained by improving photoelectron and atomic nucleus modification for photothermal conversion. The light absorption capacity is positively correlated with absorbance. The light absorption properties of SA, CPCM/SA, and AgNPs-CPCM/SA composite hydrogel sponges were studied by an ultraviolet/visible/near-infrared spectrophotometer. As shown in Figure 4a, when only SA is present, the absorbance drops sharply after a wavelength of 500 nm, and the absorbance in the medium- and long-wave regions is poor. After blending SA with CPCM, the full-wavelength absorbance of the material is stable and always maintains a high level of light absorption (85–95%), which is consistent with the characteristic of carbon-based materials having broad-spectrum and high light absorption. After AgNP loading, the light absorption decreases slightly but still remains at a high absorbance (80–90%). Combined with the stretching of O-H in the range of 3600–3200 cm^−1^ in the Fourier transform infrared absorption spectrum, it is confirmed that CPCM contains phenolic hydroxyl groups and aromatic rings. The π-π* transition of the conjugated system can strongly absorb visible light (400–760 nm), and the metal elements contained therein can form trace metal oxides or ions to assist in absorbing light of specific wavelengths by d-d transition. Combined with the increased surface roughness of CPCM/SA in the SEM image, when sunlight irradiates the surface, the roughness promotes light scattering and reflection, and the interior of CPCM/SA is more likely to absorb light, thereby improving the light absorption rate. It indicates that the loading of CPCM makes up for the light absorption capacity of the base material SA, may reduce the phase separation at the internal interface of the SA matrix, and makes the overall light absorption performance of CPCM/SA more stable through its own dispersion characteristics. The subsequent modification of AgNPs has no significant effect on the absorbance and stability of the material, and both CPCM/SA and AgNPs-CPCM/SA have high-level light absorption performance.

Figure 4b shows the heating–cooling curve of the composite hydrogel sponge within 6 min, with the first 4 min being heating, and cooling occurring after removing the light source from 5 to 6 min. The surface temperature of AgNPs- CPCM/SA rapidly rises to 69.4 °C within 4 min, with a heating rate of 10.38 °C/min, indicating that light energy can be converted into heat energy more efficiently. It is worth noting that the surface temperature of AgNPs-CPCM/SA is higher than that of CPCM/SA, which further verifies that the combination of AgNPs particles provides an excellent photothermal effect. These results show that AgNPs-CPCM/SA has excellent water absorption performance, outstanding sunlight absorption capacity, and efficient thermal localization characteristics, which are crucial for achieving efficient solar-driven interface reactions.

In the actual seawater desalination process, repeated heating and cooling will generate continuous thermal stress, which will damage the physical morphology and structure of the material. It is one of the reasons for the degradation of material performance and directly determines the actual service life of photothermal conversion materials. Therefore, a cyclic heating and cooling test was carried out eight times on AgNPs-CPCM/SA to evaluate the stability of its photothermal capacity(Figure 4). Under the same light conditions, there is no significant difference in the heating and cooling rates and the maximum achievable temperature of AgNPs-CPCM/SA. It shows excellent repeatability in terms of temperature response and can maintain good photothermal performance, eliminating the possibility that the composite hydrogel sponge has excellent initial performance but fails rapidly afterward. The combination of AgNPs and CPCM helps the composite maintain stable photothermal performance during repeated heating and cooling processes.

To further clarify the photothermal enhancement mechanism, quantitative analyses were performed from three aspects: light absorption, water state regulation, and thermal localization. The results show that coffee grounds-derived carbon material (CPCM) provides efficient broad-spectrum light absorption (85–95%). It can be inferred from the UV-Vis data that AgNPs improve the light absorption capacity of the composite material, which may be related to the localized surface plasmon resonance effect. Meanwhile, the detailed calculation of water evaporation enthalpy based on the dark-field method (Supplementary Notes S1–S3) confirms that the evaporation enthalpy test results suggest that the 3D hydrophilic network changes the existing state of water molecules, which helps to lower the energy requirement for water evaporation. It can be seen from infrared thermal images that the porous structure of the material helps to concentrate heat on the evaporation surface and reduce heat dissipation. The combined advantages of favorable light absorption, appropriate water evaporation enthalpy and heat distribution endow the composite sponge with good photothermal conversion performance.

### 3.3. Wettability

**Figure 5:**
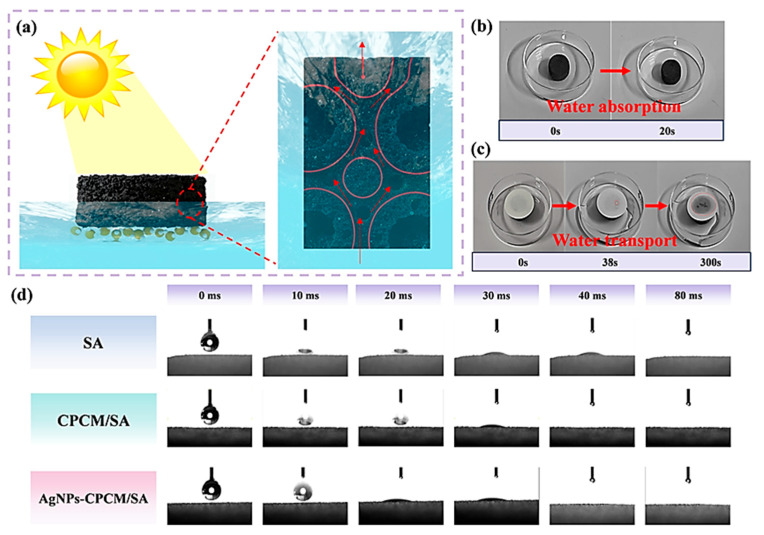
(**a**) Water transport process of AgNPs-CPCM/SA under light irradiation(The red dashed line represents the enlarged view of the material structure; the red arrows indicate the water vapor transport pathways inside the porous skeleton; the red circles mark the porous framework of the evaporator.). (**b**) Process of AgNPs-CPCM/SA absorbing 2 mL of water. (**c**) Process of 2 mL of water transporting from the bottom of AgNPs-CPCM/SA to the surface interface. (**d**) Water contact angles of SA, CPCM/SA and AgNPs-CPCM/SA, respectively.

Water transmission rate is crucial for fully supplying water to the evaporation interface and is one of the prerequisites for achieving efficient water phase change. In the light–steam conversion system, the hydrophilic and hydrophobic properties of materials affect the transmission of water molecules [6]. When a material has high hydrophilicity, a strong interaction is formed between water molecules and the material, promoting the transmission of water molecules inside the material. Therefore, water was used as the test liquid to measure the contact angle of the sample to judge the hydrophilicity of the photothermal conversion material. The wettability of different composite hydrogel sponges was evaluated by the time taken for the complete wetting and spreading of the droplet (Figure 5). There are significant differences in wettability between the matrix sponge (SA) and the composite hydrogel sponges (CPCM/SA and AgNPs-CPCM/SA). It takes 80 ms for the droplet to fully spread on the SA surface, while only 40 ms are needed for complete wetting and spreading on the CPCM/SA and AgNPs-CPCM/SA surfaces, respectively, with a substantial reduction in time. This is mainly because the water contact angle of SA is less than 30°, showing significant hydrophilic characteristics. After combining with CPCM, the surface roughness is improved, the specific surface area is increased, the contact surface with the droplet is expanded, and its hydrophilicity is enhanced. In addition, the introduction of AgNPs may increase the number of nanoscale pores, endowing AgNPs-CPCM/SA with higher capillary pressure, enabling water droplets to wet and spread on the material surface more rapidly.

AgNPs-CPCM/SA, which showed the best hydrophilicity, was selected to further verify the water transmission performance. The water absorption and the transmission speed of water inside AgNPs-CPCM/SA were evaluated by testing the time for the material to absorb water and the time for water to transmit from the bottom to the upper surface interface. At 38 s, water appeared at the surface interface, and it was completely covered after 300 s. CPCM can change the three-dimensional polymer network structure of the matrix material, affect the state of water, and further reduce the evaporation enthalpy of water. In this light–steam conversion system, light energy is absorbed and converted into heat energy, which can be utilized in situ to drive the liquid–gas phase change in water molecules transported from the water transmission channel below to the polymer network. The microstructure formed by the interaction between AgNPs and CPCM can reduce heat energy loss and ensure sufficient water supply for evaporated water.

### 3.4. Water Evaporation Performance

**Figure 6:**
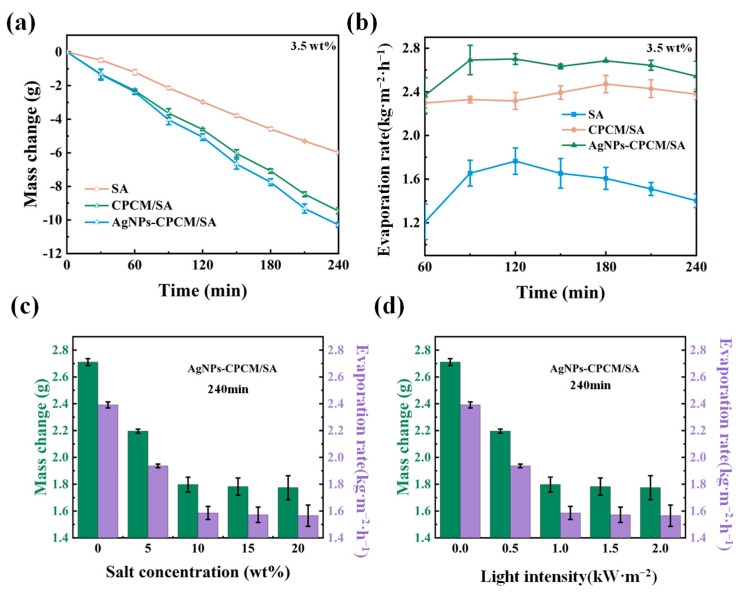
(**a**) Mass change in saltwater evaporated under one sun for 4 h by SA, CPCM/SA and AgNPs-CPCM/SA; (**b**) evaporation rate of saltwater under one sun for 4 h by SA, CPCM/SA and AgNPs-CPCM/SA; (**c**) evaporation rate and photothermal conversion efficiency of AgNPs-CPCM/SA under light intensities of 0, 0.5, 1, 1.5, and 2 suns(The green bars represent the mass change of water, and the purple bars correspond to the evaporation rate of the sample.); (**d**) evaporation rate and photothermal conversion efficiency of AgNPs-CPCM/SA in saltwater with NaCl concentrations of 0, 5, 10, 15, and 20 wt% NaCl (The green bars represent the mass change of water, and the purple bars correspond to the evaporation rate of the sample).

A simulated illumination experiment was conducted on the composite hydrogel sponge under one sun intensity in the laboratory. The water evaporation rate and mass loss varying with time during the experiment were calculated, and a handheld thermal imager was used to measure the temperature at the center of the surface interface of the material’s light absorption layer during heating and cooling within 6 min, so as to evaluate its light conversion performance and water evaporation performance. The average water evaporation rates of SA and CPCM/SA were 1.195 kg·m^−2^·h^−1^ and 1.917 kg·m^−2^·h^−1^, respectively, both higher than that of pure water (0.45 kg·m^−2^·h^−1^). The mass of AgNPs-CPCM/SA decreased by 10.17 g within 4 h, with an average water evaporation rate of 2.023 kg·m^−2^·h^−1^. The amplitude of the mass change curve in Figure 6a was mutually verified with the water evaporation rate curve in Figure 6c, indicating that the composite of AgNPs and CPCM/SA improved the water evaporation rate. The high evaporation rate of AgNPs-CPCM/SA was attributed not only to its geometric advantages but also to its unique low evaporation enthalpy. The hydrophilic network structure changes the interaction between the material and water molecules. The micro-nano pores adjust the evaporation behavior of water, and the measured evaporation enthalpy is lower than that of pure water, enabling the AgNPs-CPCM/SA evaporator to achieve a more efficient water evaporation rate. In addition, the photothermal conversion efficiency is also affected by hydrophilicity. AgNPs-CPCM/SA with good hydrophilicity has a better binding ability with water, good hydrophilicity ensures sufficient water supply at the evaporation interface, and the absorbed solar energy is well utilized for water phase transition. As a result, the photothermal conversion efficiency of the AgNPs-CPCM/SA evaporator reaches 92.32%.

To verify the photothermal conversion performance of AgNPs-CPCM/SA in different application scenarios, a salt concentration gradient experiment with 5–20 wt% was carried out. In Figure 6d, the evaporation rate of AgNPs-CPCM/SA was as high as 1.76 kg·m^−2^·h^−1^ at 5 wt%, indicating that AgNPs-CPCM/SA is suitable for offshore seawater desalination. It remained between 1.47 and 1.89 kg·m^−2^·h^−1^ at salt concentrations from 10 wt% to 20 wt%, showing the ability to realize seawater desalination in high-salinity environments such as concentrated brine and salt lake brine, which can cover most application scenarios. When the salt concentration exceeded 10 wt%, the water evaporation rate decayed, which may be due to the increase in surface tension and thermal conductivity of water at high salt concentrations, leading to increased heat loss.

To systematically evaluate the desalination capacity beyond the evaporation rate, ICP-OES was employed to quantify the residual concentrations of major cations (Na^+^, K^+^, Ca^2+^, Mg^2+^) in raw simulated seawater and condensed freshwater collected from CPCM/SA and AgNPs-CPCM/SA photothermal evaporators. All raw ion concentration data are summarized in Table S1. As listed in Table S1, both evaporators achieved extremely high rejection efficiencies over 99.9% for all four typical seawater cations. Specifically, the AgNPs-CPCM/SA sponge delivered a higher Na^+^ rejection rate (99.9990%) than the pristine CPCM/SA sample (99.9967%), which can be attributed to the enhanced electrostatic adsorption of salt ions induced by silver nanoparticles. Furthermore, we supplemented the conductivity test of condensed freshwater as an auxiliary indicator for total salinity. The ultra-low conductivity of collected water further verified the excellent salt separation performance of the composite sponges, demonstrating that the materials can effectively remove inorganic salts from seawater rather than merely accelerating water evaporation. As shown in Table S2, the feed simulated seawater exhibited high conductivity (411 μS cm^−1^) and TDS value (206 mg L^−1^). After photothermal evaporation treatment, the collected condensed water presented drastically reduced conductivity (7.35 μS cm^−1^) and ultra-low TDS (0.869 mg L^−1^). The dramatic drop in overall water conductivity and TDS strongly proves that both composite sponges can efficiently intercept most dissolved inorganic salts in seawater, consistent with the ultra-high cation rejection efficiency calculated from ICP-OES results.

### 3.5. Practical Application

**Figure 7:**
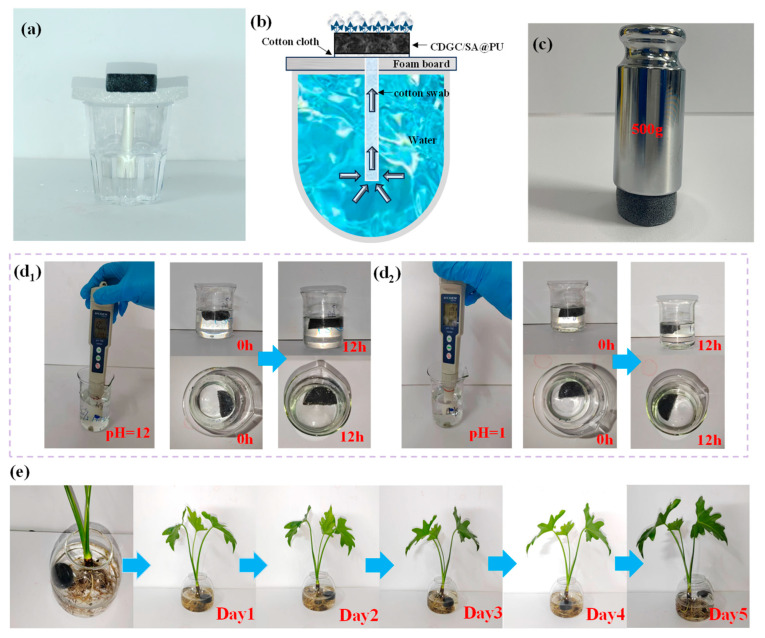
(**a**) Device for seawater desalination using AgNPs-CPCM/SA; (**b**) water evaporation process of AgNPs-CPCM/SA (The arrows indicate the transport path of water inside the cotton substrate.); (**c**) AgNPs-CPCM/SA supporting a weight of 500 g after wetting; (**d_1_**,**d_2_**) states of AgNPs-CPCM/SA at pH = 1 and pH = 12 after 0 and 12 h; (**e**) five-day co-growth process of AgNPs-CPCM/SA with plants.

Considering the potential of solar interfacial evaporation materials to be corroded or squeezed by organisms on the surface over time in practical applications, including changes in the pH value of the placement environment, seawater pressure, and extrusion by marine organisms, we evaluated the mechanical properties of AgNPs-CPCM/SA by testing its pressure-bearing capacity. As can be seen from Figure 7c, no deformation or damage occurred under a pressure of 500 g. The physical and mechanical strength of hydrogel is one of the key characteristics determining its application. The supporting effect of the porous skeleton of AgNPs-CPCM/SA improves its mechanical properties and avoids structural collapse and subsequent failure to work due to external forces.

Chemical stability is a key indicator for its application in complex aquatic environments. We tested the stability of AgNPs-CPCM/SA under strong acid and strong alkali conditions for 12 h respectively, and investigated the changes in the morphology, dispersibility and integrity of the material. It can be observed from Figure 7d that the physical morphology of AgNPs-CPCM/SA did not change after 12 h, with no dissolution, delamination, damage or other phenomena, and the overall structural integrity was well maintained.

To further verify the biosafety of AgNPs-CPCM/SA, AgNPs-CPCM/SA was placed in hydroponic plant culture bottles to compare the plant growth status. As can be seen from Figure 7e, the leaves of hydroponic plants remained bright green during the culture period, and the overall growth status was good without adverse phenomena such as withering and yellowing.

**Figure 8:**
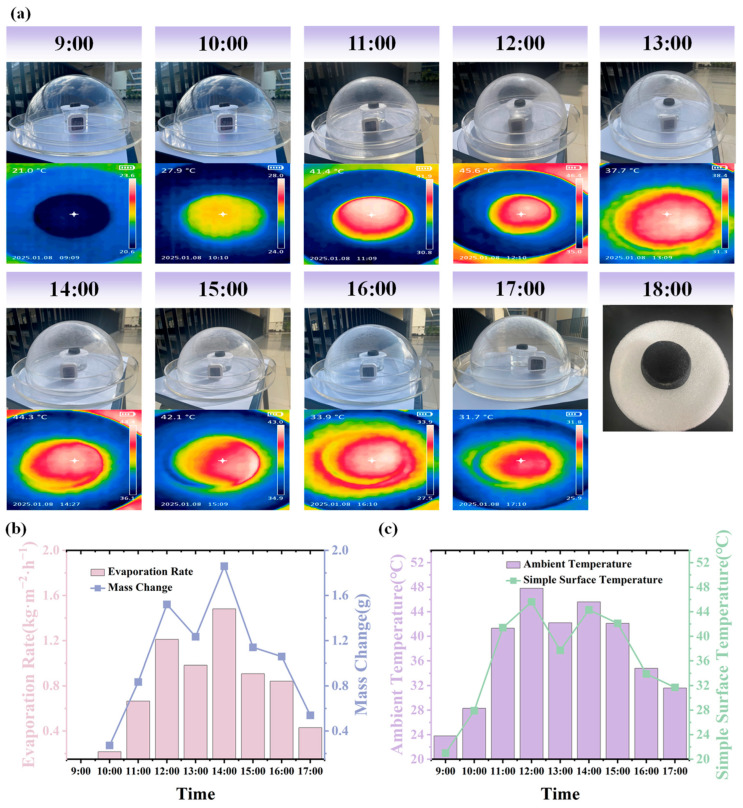
(**a**) Infrared thermal images of AgNPs-CPCM/SA under one sun for 8 h and the material state after the water evaporation experiment; (**b**) evaporation rate and mass change in AgNPs-CPCM/SA under one sun for 8 h; (**c**) curves of ambient temperature and central surface temperature of AgNPs-CPCM/SA under one sun for 8 h.

An 8 h water evaporation experiment was conducted under natural light, with the hourly water evaporation rate, weather conditions, surface temperature and ambient temperature recorded. The temperature response characteristics of the photothermal material were observed from the infrared thermal images under outdoor natural light in Figure 8a. From 9:00 to 14:00, the high-temperature area on the surface interface of AgNPs-CPCM/SA gradually expanded with the increase in solar irradiance intensity, and the temperature rose continuously, reaching a peak of 44.4 °C at 14:00, which corresponded to the maximum solar irradiance intensity at noon. From 14:00 to 18:00, as the solar irradiance intensity weakened, the proportion of the high-temperature area on the material surface decreased, and the maximum temperature dropped. These results indicate that AgNPs-CPCM/SA has an excellent temperature response to natural solar irradiance and can effectively increase the surface temperature through photothermal conversion, providing thermal energy support for the seawater desalination process.

Combined with the variation in water evaporation rate in Figure 8b, it is shown that the water evaporation rate of AgNPs-CPCM/SA in practical applications is dependent on solar irradiance intensity, and the material can achieve efficient water evaporation performance under natural light conditions, with the peak period of its evaporation rate consistent with the strongest period of solar irradiance. In addition, the material surface temperature always follows the same variation trend as the ambient temperature, demonstrating that AgNPs-CPCM/SA maintains excellent thermal localization in practical applications. It can concentrate heat in the evaporation area, reduce heat loss to the environment, and improve the overall energy utilization efficiency.

During the continuous evaporation of seawater, salt accumulation on the surface interface of photothermal conversion materials leads to the precipitation of solid salt crystals on the photothermal conversion interface, which in turn affects the material’s light absorption, hinders water transport and evaporation, and reduces the evaporation rate. The problem of surface salt accumulation of photothermal conversion materials is a major challenge for continuous water evaporation in solar interfacial evaporation. To address this challenge, scholars have proposed many anti-salt strategies, including the design of special pore structures to enhance the diffusion and reflux of salt ions into the bulk water, thus avoiding salt crystallization. Figure 8c shows the state of the photothermal conversion material after the water evaporation experiment, from which it can be seen that no obvious salt crystallization appeared on the surface interface of the hydrogel sponge after more than 8 h of continuous operation. The highly interconnected pore structure enhances the diffusion and reflux of salt ions into water, provides more direct migration paths for salt ions, reduces diffusion resistance, and promotes the rapid and uniform distribution and reflux of ions in the solution.

To evaluate the economic practicability of AgNPs-CPCM/SA, we calculated its total preparation cost, and the detailed cost accounting is listed in Table 1. To further demonstrate the outstanding photothermal evaporation performance of the composite, we compared its evaporation rate with other reported solar evaporators, and the results are summarized in Table 2.

### 3.6. Photocatalytic Performance

**Figure 9:**
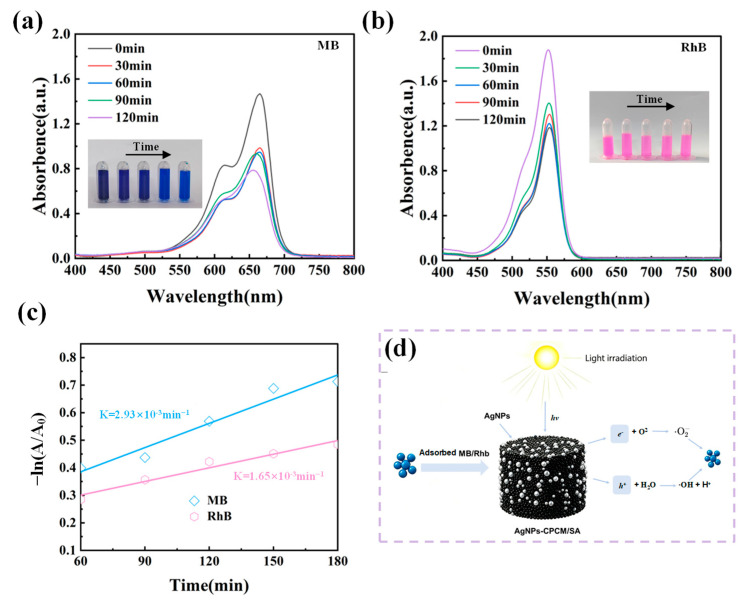
Time-resolved UV-Vis absorption spectra for (**a**) methylene blue and (**b**) rhodamine B treated by AgNPs-CPCM/SA under visible light irradiation; (**c**) first-order kinetic plots for visible-light-driven degradation of MB(blue line) and RhB(pink line); (**d**) schematic illustration for the photocatalytic degradation mechanism of MB over AgNPs-CPCM/SA under light irradiation.

To further explore the application potential of AgNPs-CPCM/SA in water pollution treatment, methylene blue (MB) and rhodamine B (RhB) with concentration of 20 mg/L were selected as simulated pollutants. The photoresponsive removal performance under visible light irradiation was investigated via UV-Vis absorption spectroscopy. Figure 9a,b display the time-resolved UV-Vis spectra of MB and RhB in the presence of AgNPs-CPCM/SA. A characteristic absorption peak of MB is located at 664 nm. As irradiation time prolonged, the absorbance intensity gradually declined with a slight blue shift, and the dark blue color of solution faded correspondingly. The blue shift in the MB characteristic peak is caused by the destruction of conjugated structure and the generation of degradation intermediates during photocatalytic reaction, which indicates the effective degradation of dye molecules. It is demonstrated that the concentration of MB decreased during the reaction, and AgNPs-CPCM/SA possessed favorable removal capacity for MB under visible light. Similarly, the characteristic peak of RhB at 554 nm decreased without peak shift, accompanied by the fading of pink solution color, proving the effective removal of RhB. No additional absorption peaks were observed throughout the reaction process, revealing that no stable colored intermediates were produced.

Under light irradiation, photogenerated electrons enriched on the surface of AgNPs can reduce dissolved oxygen to produce superoxide radicals; the holes trapped on the carbon matrix can oxidize water molecules to generate hydroxyl radicals [35]. As shown in Figure 9d, Ag nanoparticles exert dual effects on photocatalytic activity under continuous light irradiation. Benefiting from the localized surface plasmon resonance (LSPR) of AgNPs, the separation of photoinduced electrons and holes is greatly accelerated, which promotes the generation of ·O_2_^−^ and ·OH radicals and thus enhances the visible-light degradation capacity toward organic dyes [36]. The photocatalytic degradation kinetic curves of MB and RhB reveal that the absorbance of methylene blue continuously decreases with prolonged illumination time, indicating the gradual oxidative decomposition of organic dye molecules. To quantitatively evaluate the photodegradation kinetics of MB and RhB, the pseudo-first-order kinetic model was adopted to fit the experimental data, and the corresponding kinetic plots are presented in Figure 9c. The apparent rate constant k was determined from the slope of each linear fitting curve. The k value of MB surpasses that of RhB, which demonstrates that AgNPs-CPCM/SA delivers enhanced visible-light photocatalytic activity for the elimination of methylene blue relative to rhodamine B.

## 4. Conclusions

In summary, a composite hydrogel sponge with high light absorption performance, low energy loss and reduced water evaporation enthalpy for solar interfacial water evaporation was prepared by depositing silver nanoparticles on a porous carbon-based framework system via a simple physical adsorption method. In the evaporator, SA hydrogel with an ion-cross-linked three-dimensional network structure was used as the matrix material, which ensured the repulsion of multivalent ions based on the distribution of carboxylate anions. The superhydrophilic properties and micro-nanoscale pores of AgNPs-CPCM/SA provided convenient channels for the transport of water molecules and the escape of water vapor. The combination of CPCM and AgNPs improved the light absorption and photothermal conversion efficiency of AgNPs-CPCM/SA, providing a stable supply of thermal energy for water phase change. As a result, the interfacial water evaporation rate of AgNPs-CPCM/SA reached 2.023 kg·m^−2^·h^−1^ with a water evaporation efficiency of 92.32%, demonstrating great application potential in seawater desalination. In addition, the results of practical application tests confirmed that AgNPs-CPCM/SA possesses mechanical stability and biological safety, making it a promising light-to-vapor conversion composite material with broad application prospects. Meanwhile, the as-prepared composite possesses excellent photocatalytic capability. It can efficiently eliminate common organic dyes including methylene blue and rhodamine B under visible-light irradiation, with no stable colored intermediates generated during the reaction. This material integrates seawater desalination and organic wastewater purification functions, which broadens its application prospects in water environmental treatment.

## Figures and Tables

**Table 1:** Cost accounting.

Category	Quantity Used	Unit	Unit Price (USD)	Total Price (USD)
SA	4	g	0.006	0.024
Coffee powder	2	g	0.005	0.010
Lignin	0.5	mL	0.244	0.122
AgNO_3_	0.2	mL	1.970	0.394
NH_3_·H_2_O	5	mL	0.006	0.030
CaCl_2_	2	g	0.006	0.012
Carbonization cost (Nitrogen)	0.5	MPa	1.95	0.975
Depreciation cost of other equipment	-	-	2	2
Other laboratory consumables	-	-	3	3
Electricity	10	kW/h	0.15	1.5
Total	8.067			

**Table 2:** Comparison with recently reported solar evaporators.

Number	Material	Evaporation Rate (kg·m^−2^·h^−1^)	References
1	PDAppy-S1.5	1.85	[30]
2	Cd-MOF	1.78	[31]
3	(FeCoNiCrCu)O	1.88	[32]
4	rGO/CNF	1.65	[33]
5	PPy@D-SCG	1.54	[34]
6	AgNPs-CPCM/SA	2.02	This work

## Data Availability

Data will be made available on request.

## Supplementary Materials

The following supporting information can be downloaded at: https://www.mdpi.com/article/10.3390/nano16140864/s1. Figure S1. Water evaporation rate of AgNPs-CPCM/SA under one sun irradiation for four consecutive days; Figure S2. Digital photos of AgNPs-CPCM/SA evaporation under one sun irradiation for three consecutive days; Figure S3. Apparent porosity (ε) of SA, CPCM/SA, AgNPs-CPCM/SA; Table S1. Cation concentrations of feed seawater and freshwater produced by CPCM/SA and AgNPs-CPCM/SA sponges; Table S2. TDS, conductivity and salinity of initial simulated seawater and condensed freshwater collected from AgNPs-CPCM/SA; Table S3. Static water immersion test data of porous sponge samples.
