# Supplementary material for: A Three-Dimensional Porous Ag/C/Sodium Alginate@Polyurethane Sponge for Efficient Solar-Driven Seawater Desalination
Source: Nanomaterials (Basel). 2026 Jul 14;16(14):864. doi: 10.3390/nano16140864 (PMC13415617; doi:10.3390/nano16140864)
Supplement: Supplementary file 1 [file nanomaterials-16-00864-s001.zip › nanomaterials-4338206-supplementary.pdf]

Supporting information

A Three-Dimensional Porous Ag/C/Sodium Alginate@Polyurethane Sponge for Efficient Solar-Driven Seawater Desalination

Yingying Yue [1,*], Rou Zeng [1], Yingfei Wang [1], Jiaqi Hu [1], Chengxin Zhang [1], Yu Li [1], Weijie Wei [2], Wubo Wan [1], Zaoxi Li [1] and Yaqin Shi [1,*]

[1] Yazhou Bay Innovation Institute, College of Food Science and Engineering, Hainan Tropical Ocean University, Sanya 572022, China;
[2] School of Chemistry and Chemical Engineering, Hainan University, Haikou 570228, China;
* Correspondence: yueyy@hntou.edu.cn (Y.Y.); shiyaqincelia@163.com (Y.S.); Tel.: +86-18117704244 (Y.Y.); +86-13006069810 (Y.S.)

Supplementary Note S1: The calculation for evaporation enthalpy

The dark-field evaporation enthalpy measurement adopted in this work has been widely reported and verified in a large number of photothermal evaporation literatures. The core energy conservation assumption (pure water and composite evaporator share identical energy dissipation under the same closed dark environment) is strictly guaranteed by consistent experimental conditions (closed container, relative humidity 50±5%, 25 °C, 12 h dark standing). All dark evaporation data were averaged from three parallel replicates before calculation.

In this study, the dark-field evaporation method was used to calculate the evaporation enthalpy of water. Generally, the following assumption is made: in the same closed dark environment, the energy of the pure water system and the composite hydrogel evaporator obeys the law of conservation of energy. The composite hydrogel evaporator and pure water with the same area were placed in a closed container with a relative humidity of approximately 50±5%. The experiment was carried out at room temperature (25 °C) for 12 h in darkness. The evaporation amounts of the two systems were measured, and the evaporation enthalpy was obtained according to **Equation S1**, where $\dot{m}_{water}$ and $\dot{m}_{eva}$ are the evaporation rates of pure water and the composite hydrogel evaporator at 25 °C(kg·m^{-2}·h^{-1}). $h_{ee,water}$ and $h_{ee,eva}$ are the evaporation enthalpies of pure water and the composite hydrogel evaporator at 25 °C(kJ·kg^{-1}).

$$\dot{m}_{water} h_{ee,water} = \dot{m}_{eva} h_{ee,eva} \tag{S1}$$

The calculation parameters and results are shown below:

	$\dot{m}_{25°C}$(kg·m^{-2}·h^{-1})	$h_{ee,25°C}$(kJ·kg^{-1})
Water	0.0086	2442.0
CPCM/SA	0.0127	1653.7

All dark evaporation data were averaged from three parallel replicates before substitution into Equation S1.

The evaporation enthalpy at different temperatures was estimated. The evaporation enthalpy of pure water at different temperatures was calculated by **Equation S2**, where $h_{ee,water,T_1}$ is the evaporation enthalpy of pure water at T_1(kJ·kg^{-1}). T_1 is the steady-state temperature of the evaporator and pure water under illumination(K). $C_{p,l}$ is the specific heat capacity of liquid water(4.2kJ·K^{-1} kg^{-1}). $h_{ee,water,100°C}$ is the evaporation enthalpy of water at 100 °C(2257kJ·kg^{-1}). $C_{p,g}$ is the specific heat capacity of gaseous water(kJ·K^{-1}·kg^{-1}). calculated by **Equation S3**, where R is the universal gas constant(8.314kJ·K^{-1}·mol^{-1}), M

is the molar mass of water(18.02g·mol-1). $h_{ee,water,100°C}$is the evaporation enthalpy of water at 100 °C (2257 kJ·kg−1); $C_{p,g}$ is the specific heat capacity of gaseous water (kJ·K−1·kg−1).

$$h_{ee,water,T_1} = \int_{T_1}^{100°C} C_{p,l}dT + h_{ee,water,100°C} + \int_{100°C}^{T_1} C_{p,g}dT \qquad (S2)$$

$$C_{p,g} = (3.470 + 1.45 \times 10^{-3} \times T_1 + 0.121 \times 10^5 \times T_1^{-2})RM^{-1} \qquad (S3)$$

Equation S4 was obtained by transforming **Equations S1** and **S2**. The evaporation enthalpy of the membrane evaporator at different temperatures can be estimated by **Equation S4**.

$$h_{ee,eva,T_1} = \frac{\dot{m}_{water}}{\dot{m}_{eva}} h_{ee,water,T_1} \qquad (S4)$$

The calculation parameters and results are shown below:

**Evaporators**	***T1* (K)**	$C_{p,g}$	$h_{ee,water,T_1}$ **(kJ·kg-1)**	h_{ee,eva,T_1} **(kJ·kg-1)**
Water	301.25	1.906	2424.9	2424.9
AgNPs-CPCM/SA	342.55	1.892	2386.3	1465.2

Temperature and enthalpy parameters are calculated based on three parallel dark-field test datasets.

Supplementary Note S2: The calculation for enthalpy change.

The enthalpy change from water to vapor includes the sensible heat of evaporation and the latent heat of evaporation, as shown in Equation S5. Where h_{lv,eva,T_1} is the enthalpy change from water to vapor(kJ/kg). h_{ee,eva,T_1} is the evaporation enthalpy (kJ/kg). h_{sh,eva,T_1} is the sensible heat of evaporation(kJ/kg). The sensible heat of evaporation can be calculated by **Equation S6**, where T_0 is the initial temperature of the membrane evaporator surface, $T1$ is the steady-state temperature of the membrane evaporator surface under illumination, and $C_{p,l}$ is the specific heat capacity of liquid water (4.2 kJ K−1 kg−1).

$$h_{lv,eva,T_1} = h_{ee,eva,T_1} + h_{sh,eva,T_1} \qquad (S5)$$

$$h_{sh,eva,T_1} = \int_{T_0}^{T_1} C_{p,l}dT \qquad (S6)$$

The calculation parameters and results are shown below:

**Evaporators**	***T0* (K)**	***T1* (K)**	h_{sh,eva,T_1} **(kJ·kg-1)**	h_{lv,eva,T_1} **(kJ·kg-1)**
AgNPs-CPCM/SA	301.05	342.55	174.3	1639.5

Steady-state temperature and enthalpy data are averaged values of three parallel light irradiation tests.

Supplementary Note S3: Calculation of evaporation efficiency.

The evaporation rate used in **Equation S7** is the net evaporation rate after subtracting spontaneous dark evaporation measured from blank dark-field experiments. Since brine evaporation was adopted in this experiment, the evaporation efficiency of the membrane evaporator was calculated by Equation S7. Where $\dot{m}$ is the evaporation rate (kg m-2 h -1). h_{lv} is the enthalpy change from water to vapor(kJ·kg-1). W_{least} epresents the theoretical minimum energy required to separate the salt solution into solid salt and pure water. The energy consumption for separating a 35 g/kg NaCl solution into pure water and solid salt is 10.75 kJ/kg, which is equivalent to 10.39 kJ/kg for pure water production (used as the approximate value for 35 g/L NaCl solution).

I is the solar irradiation intensity supplied to the device per hour(1kW/m2 h).

$$\eta = \frac{\dot{m} \cdot (h_{lv} + W_{least})}{I} \qquad (S7)$$

Evaporators	$\dot{m}$（kg m^{-2} h^{-1}）	$h_{lv,}$（kJ kg^{-1}）	W_{least}（kJ kg^{-1}）	η（%）
AGNPS-CPCM/SA	2.023	1639.5	10.39	92.32

Evaporation data are averaged from three parallel four-day continuous irradiation experiments; error bars in Figure S1 correspond to standard deviation of three parallel samples.

Supplementary Note S4: Calculation of evaporation efficiency.

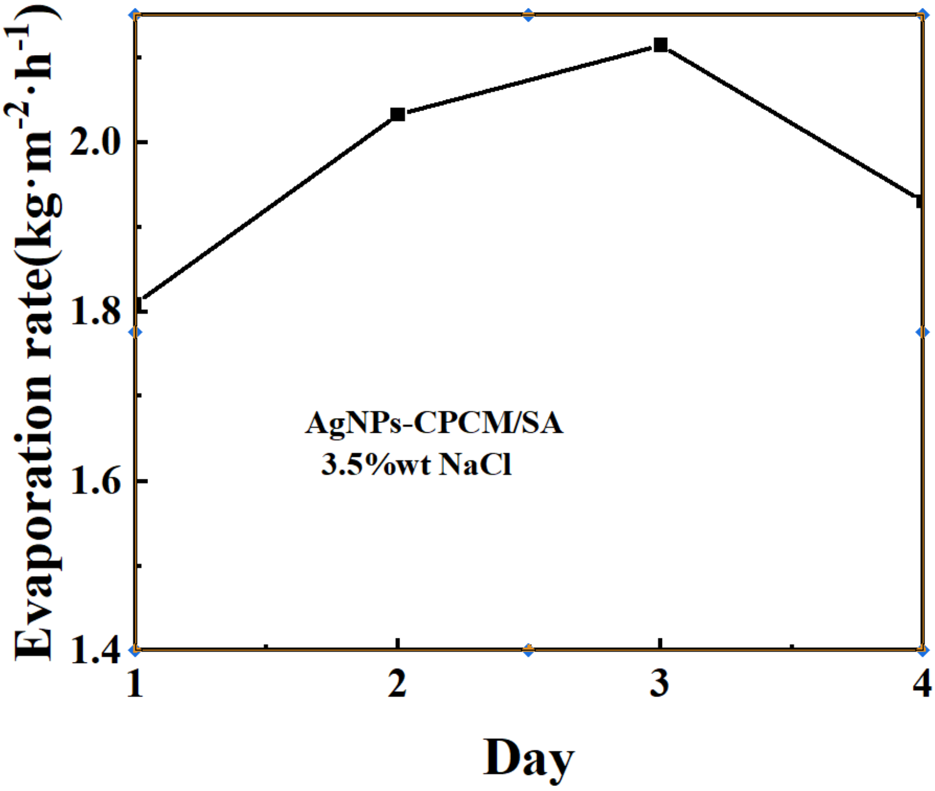

Figure S1. Water evaporation rate of AgNPs-CPCM/SA under one sun irradiation for four consecutive days.

Supplementary Note S5: Calculation of evaporation efficiency.

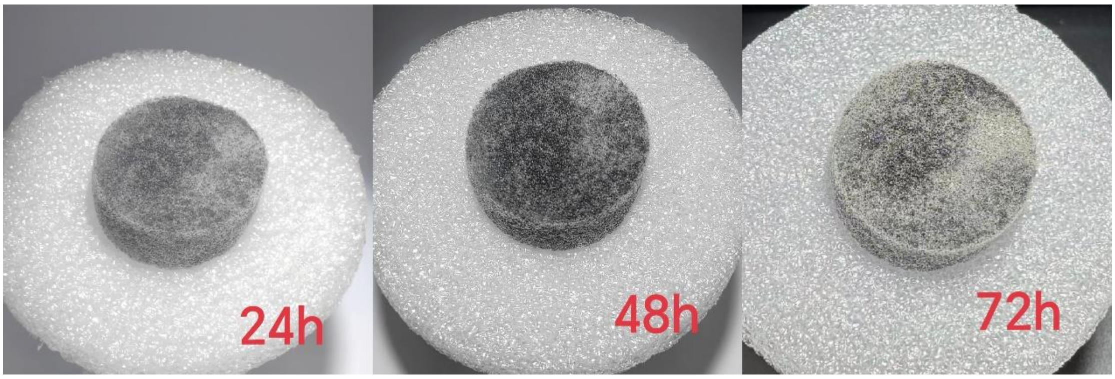

Figure S2. Digital photos of AgNPs-CPCM/SA evaporation under one sun irradiation for three consecutive days.

Supplementary Note S6: ion rejection efficiency.

The ion rejection efficiency was calculated by the **formula(S8)**:

$$R = \frac{100(C_{feed} - C_{freshwater})}{C_{feed}} \quad \text{(S8)}$$

C_{feed} and $C_{freshwater}$ are the cation concentrations of initial simulated seawater and condensed freshwater, respectively.

Table S1. Cation concentrations of feed seawater and freshwater produced by CPCM/SA and AgNPs-CPCM/SA sponges.

Cation	C_{feed} (mg/L)	$C_{CPCM/SA}$ (mg/L)	$R_{CPCM/SA}$(%)	$C_{AgNPs\text{-}CPCM/SA}$ (mg/L)	$R_{AgNPs\text{-}CPCM/SA}$(%)
Mg^{2+}	1417	0.021	99.9985	0.044	99.9969
Ca^{2+}	462.8	0.014	99.997	0.423	99.9086
K^{+}	612.18	0.121	99.9802	0.148	99.9758
Na^{+}	13432.32	0.443	99.9967	0.131	99.999

C_{feed} represents the cation concentration of initial simulated seawater; $C_{CPCM/SA}$ and $C_{AgNPs\text{-}CPCM/SA}$ refer to ion concentrations of freshwater collected from CPCM/SA and AgNPs-CPCM/SA photothermal evaporators, respectively.

Supplementary Note S7: TDS, conductivity and salinity.

Table S2. TDS, conductivity and salinity of initial simulated seawater and condensed freshwater collected from AgNPs-CPCM/SA.

Feed TDS (mg L^{-1})	Feed conductivity ($\mu S\ cm^{-1}$)	Feed salinity	Produced water TDS (mg L^{-1})	Produced water conductivity ($\mu S\ cm^{-1}$)	Produced water salinity
869	735	248	206	411	ND

Supplementary Note S8: overall pore volume fraction.

The static water immersion method was adopted to evaluate the overall porosity of the porous sponge samples. Firstly, the mass of fully dried sponge was recorded as m_1. The dried sponge was completely immersed in deionized water under atmospheric pressure until the internal pores reached water saturation. After gently wiping off free water attached to the sample surface with filter paper, the mass of water-saturated sponge was weighed and marked as m_2. The density of pure water ρ was taken as 1.0g/cm^3, and V represented the apparent bulk volume of the sponge block.

The overall pore volume fraction of the sponge was calculated by static water absorption method:

$$\varepsilon = \frac{m_2 - m_1}{\rho V} \quad \text{(S9)}$$

where m_1 and m_2 are the mass of dried and water-saturated sponge, respectively; ρ is the density of deionized water (1.0g/cm^3); V is the apparent bulk volume of the sponge sample(V=0.942cm^3).

Table S3. Static water immersion test data of porous sponge samples.

Sample	m1 (g)	m2 (g)	m2–m1 (g)	ε

SA-1	0.33	17.13	16.8	17.84
SA-2	0.34	17.29	16.96	18
SA-3	0.33	17.28	16.95	17.99
CPCM/SA-1	2.65	15.42	12.77	13.55
CPCM/SA-2	6.24	14.36	8.12	8.62
CPCM/SA-3	5.98	13.97	8	8.49
AgNPs-CPCM/SA-1	2.13	13.31	11.18	11.87
AgNPs-CPCM/SA-2	3.64	16.95	13.31	14.13
AgNPs-CPCM/SA-3	3.67	14.03	10.36	11

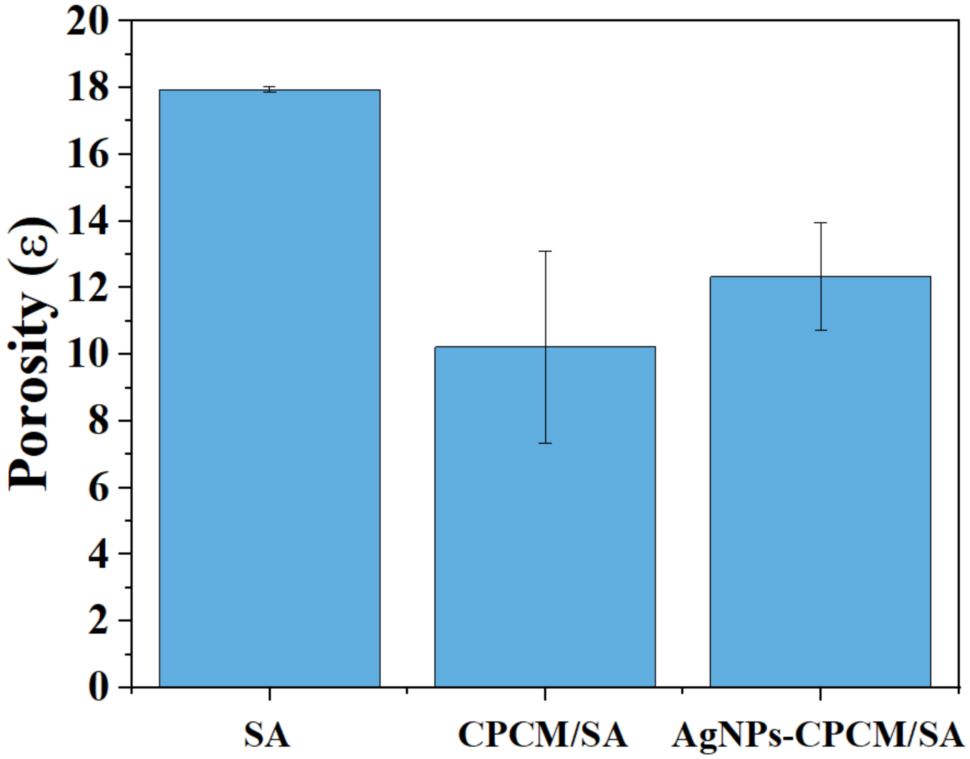

Figure S3. Apparent porosity (ε) of SA、CPCM/SA、AgNPs-CPCM/SA.
